# Closing the psychological treatment and mental health literacy gaps using ResilienceNhope, an evidence-based text and email messaging innovative program

**DOI:** 10.1192/j.eurpsy.2023.1809

**Published:** 2023-07-19

**Authors:** B. Agyapong, R. Shalaby, Y. Wei, V. I. Agyapong

**Affiliations:** Department of Psychiatry, University of Alberta, Edmonton, Canada

## Abstract

**Introduction:**

There is a high prevalence of stress, anxiety, depression, and substance use disorders in college students globally. Financial stressors, course workload, peer pressure and other personal, family, and societal stressors contribute to the high incidence of mental disorders among college students. Despite the high prevalence of mental disorders in college students, barriers such as lack of mental health literacy, stigma of mental health, inadequate numbers of mental health counsellors and clinical psychologists supporting students in colleges in both low- and high-income countries, financial and geographical barriers often hinder college students from accessing the needed mental supports.

**Objectives:**

In this article, we provide a perspective on the ResilienceNHope program, an evidence-based text and email messaging innovation to close the psychological treatment gap and improve the mental health literacy of university and college students.

**Methods:**

Review of literature. There is increasing evidence on the effectiveness and feasibility of mobile technology in health promotion and closing psychological treatment gaps. College students are well adapted to the use of mobile technology, particularly text and email messaging daily, which presents a unique opportunity for an innovative way to offer support for their mental health.

**Results:**

There’s evidence to support the findings that ResilienceNHope program, which involves the use of text and email messaging is an innovative tool which can be adopted to close the psychological treatment gap and improve the mental health literacy of university and college students

**Conclusions:**

Supportive text messaging can be adopted to help support and improve the mental health of university and college students.

**Disclosure of Interest:**

None Declared

